# Seasonal Stability of SARS-CoV-2 in Biological Fluids

**DOI:** 10.3390/pathogens10050540

**Published:** 2021-04-30

**Authors:** Taeyong Kwon, Natasha N. Gaudreault, Juergen A. Richt

**Affiliations:** Department of Diagnostic Medicine/Pathobiology, College of Veterinary Medicine, Kansas State University, Manhattan, KS 66506, USA; tykwon@vet.k-state.edu (T.K.); nng5757@vet.k-state.edu (N.N.G.)

**Keywords:** biological fluid, fomite, half-life, human, SARS-CoV-2, surface, virus stability

## Abstract

The transmission of SARS-CoV-2 occurs by close contact with infected persons through droplets, the inhalation of infectious aerosols, and the exposure to contaminated surfaces. Previously, we determined the virus stability on different types of surfaces under indoor and seasonal climatic conditions. SARS-CoV-2 survived the longest on surfaces under winter conditions, followed by spring/fall and summer conditions, suggesting the seasonal pattern of stability on surfaces. However, under natural conditions, the virus is secreted in various biological fluids from infected humans. In this respect, it remains unclear how long the virus survives in various types of biological fluids. This study explores SARS-CoV-2 stability in virus-spiked human biological fluids under different environmental conditions by determining the virus half-life. The virus was stable for up to 21 days in nasal mucus, sputum, saliva, tear, urine, blood, and semen; it remained infectious significantly longer under winter and spring/fall conditions than under summer conditions. In contrast, the virus was only stable up to 24 h in feces and breast milk. These findings demonstrate the potential risk of infectious biological fluids in SARS-CoV-2 transmission and have implications for its seasonality.

## 1. Introduction

Since its first emergence in China, SARS-CoV-2 has rapidly spread worldwide and poses a tremendous threat to global public health and the economy. The efficient transmission of SARS-CoV-2 is primarily mediated through close contact with infected individuals who shed respiratory droplets when exhaling, talking, sneezing, and coughing [1]. The expelled droplets span a wide size spectrum, but the majority of them are generally considered >5 μm in diameter. In contrast, in airborne transmission, smaller droplets, size ≤5 μm, evaporate quickly to generate infectious aerosols that can remain suspended in air for several hours and travel over a long distance [2]. A potential third route of transmission may be exposure to the infectious virus on surfaces. Large droplets settle down to the ground or surfaces within a limited distance and contaminate the environment with the virus. The infectious virus can survive for several days on various types of surfaces and the half-life is the longest under winter conditions, followed by spring/fall and summer conditions, suggesting a seasonal pattern of virus survival on contaminated surfaces [3,4]. Oro-fecal/naso-fecal, bloodborne, mother-to-child, and sexual transmissions have also been proposed, but their roles still remain unclear.

Scientists have investigated the presence of SARS-CoV-2 in biological fluids, since they serve as a potential source of infection and as clinical specimens for diagnostics. Nasal mucus, sputum, and saliva are major components to produce the respiratory droplets by which the transmission of SARS-CoV-2 predominantly occurs. Ocular changes and conjunctivitis are one of the clinical manifestations of SARS-CoV-2 infection in humans, and the virus can be detected and isolated in tears and ocular swabs [5]. In rare cases, infectious virus was isolated in the urine [6] and feces [7,8,9] of COVID-19 patients, and these findings suggest a possible route of oro-fecal/naso-fecal transmission of SARS-CoV-2 and even transmission through the wastewater system. Several studies reported the detection of SARS-CoV-2 RNA in blood [10,11], breast milk [12,13], and semen [14], which might suggest bloodborne, mother-to-child, and sexual transmission, respectively. Therefore, we evaluated SARS-CoV-2 stability in liquid and surface settings of virus-spiked human biological fluids under indoor and different seasonal conditions.

## 2. Results

**Figure 1:**
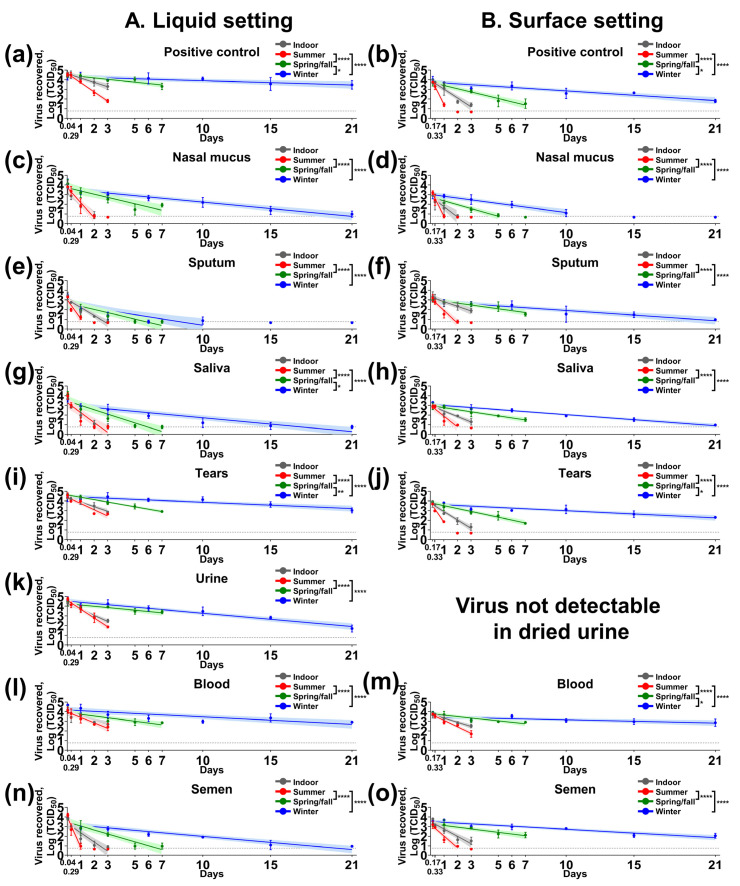
SARS-CoV-2 stability in medium, human nasal mucus, sputum, saliva, tears, urine, blood, and semen under indoor and three seasonal conditions. The mixture of the virus (5 × 10^4^ 50% tissue culture infectious dose [TCID_50_]) and each biological fluid was placed in (**A**) sealed tubes for the liquid setting (**a**,**c**,**e**,**g**,**i**,**k**,**l**,**n**) or (**B**) on stainless steel for the surface setting (**b**,**d**,**f**,**h**,**j**,**m**,**o**). The mixture was dried for 4 h inside a biosafety cabinet for the surface setting. The tubes and stainless steel were incubated under indoor (gray), summer (red), spring/fall (green), and winter (blue) conditions. At each time point, infectious virus was recovered in 2 mL medium, filtered through a 0.45 μm syringe filter, and titrated on Vero E6 cells. Virus titers were log-transformed, and a simple linear regression model was determined in Prism 9, Graphpad. Virus titers are expressed as mean log_10_ TCID_50_ ± standard deviation at each time point; the solid lines and shaded areas represent a best-fit line and 95% confidence interval of the linear regression model under each condition. The dashed line represents the limit of detection, 10^0.767^ TCID_50_, for the virus isolation assay. Statistical analysis using ANOVA and subsequent Tukey’s adjustment was performed to determine seasonal difference of t_1/2_ half-life of SARS-CoV-2 in summer, spring/fall, and winter conditions. Adjusted p values for significance are marked: * (*p* < 0.05), ** (*p* < 0.01), and **** (*p* < 0.0001). On the *x*-axis, 0.04, 0.17, 0.29 and 0.33 days are equal to 1, 4, 7, and 8 h, respectively.

We estimated SARS-CoV-2 half-life (t_1/2_) values in human nasal mucus, sputum, saliva, tears, blood, and semen under both liquid and a non-porous surface setting (steel), as well as human urine in liquid only, by spiking 5 × 10^4^ 50% tissue culture infectious dose (TCID_50_) of SARS-CoV-2 into biological fluids from healthy donors. Under indoor conditions (21 °C/60% relative humidity (RH)), t_1/2_ values ranged from 5.23 to 16.74 h in liquid and from 6.77 to 16.57 h on the steel surface (Table 1). We found t_1/2_ values ranging from 2.3–12.57 h and 2.58–10.75 h in liquid and surface settings, respectively, under summer conditions (25 °C/70% RH). The incubation under spring/fall conditions (13 °C/66% RH) resulted in slower virus decay and longer t_1/2_ values, resulting in 15.98–54.34 h in liquid and 18.15–48.4 h for the surface setting. The longest virus survival was observed under winter conditions (5 °C/75% RH), where t_1/2_ values were 33.37–121.83 h in liquid and 38.55–235.18 h for the steel surface setting. Statistical analysis on liquid and surface settings showed that the t_1/2_ values under summer conditions were significantly different from either the spring/fall or winter conditions (*p* < 0.0001) for human nasal mucus, sputum, saliva, tears, blood, and semen, and for human urine in liquid (Figure 1). In addition, we found significant differences between spring/fall and winter conditions for human saliva (*p* = 0.0331) and tears (*p* = 0.0029) in liquid, and for tears (*p* = 0.0146) and blood (*p* = 0.0202) under the surface setting. SARS-CoV-2 was significantly more stable in the liquid setting than surface setting for nasal mucus under summer conditions, and it was more stable in the liquid setting than the surface setting for tears under indoor and summer conditions (Table 1). In contrast, the t_1/2_ values were significantly higher on the surface than liquid for sputum under indoor, spring/fall, and winter, for saliva under indoor and spring/fall, for blood under winter, and for semen under summer, spring/fall, and winter conditions.

**Figure 2:**
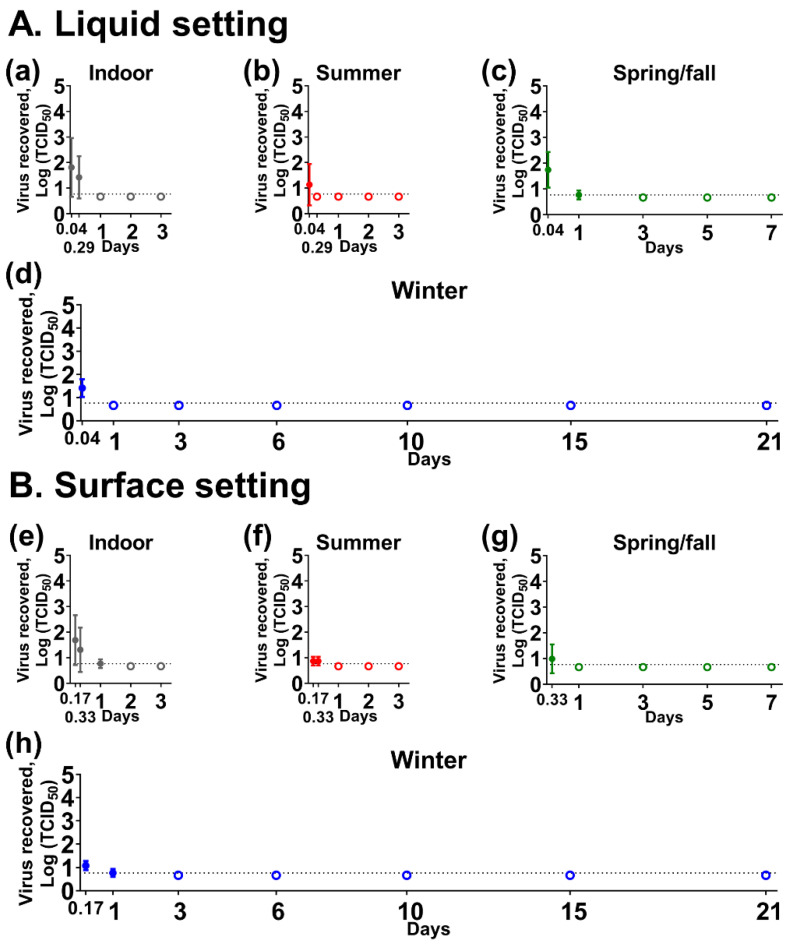
SARS-CoV-2 stability in human breast milk. A mixture of the virus (5 × 10^4^ 50% tissue culture infectious dose [TCID_50_]) and breast milk was placed (**A**) in sealed tubes for the liquid setting (**a**–**d**) or (**B**) on stainless steel for the surface setting (**e**–**h**) and incubated under indoor (**a**,**e**), summer (**b**,**f**), spring/fall (**c**,**g**), and winter (**d**,**h**) conditions. Virus titer was expressed as mean log_10_ TCID_50_ ± standard deviation at each time point. The dashed line represents a limit of detection, 10^0.767^ TCID_50_, for the virus isolation assay. Empty dots represent negative results tested in triplicate. On the *x*-axis, 0.04, 0.17, 0.29, and 0.33 days are equal to 1, 4, 7, and 8 h, respectively.

However, the virus was unstable under both liquid and surface settings in human feces, fecal suspension, and breast milk, as well as in urine under the surface setting. In human fecal suspension, low virus titers were found at 1-h post-contamination (hpc) in liquid under indoor (0.978 ± 0.317 log TCID_50_) and summer conditions (0.767 ± 0.174 log TCID_50_). No infectious virus was recovered from human feces for either setting, or from fecal suspension and urine on the steel surface. In both settings of human breast milk, infectious virus was detected up to 1-day post-contamination (dpc), in which virus titers ranged from 0.767 ± 0.174 to 1.812 ± 1.151 log TCID_50_ (Figure 2).

## 3. Discussion

Extended stability under winter seasonal conditions are one of the intrinsic characteristics of SARS-CoV-2, which could potentially contribute to spread of the virus through contaminated surfaces and enhance the transmission in cold weather. Earlier studies have investigated the virus stability by contaminating various surfaces with cell culture-derived virus inoculum [3,4,15]. The virus remained infectious on various surfaces for several days under indoor conditions, and its survival was significantly longer under winter climatic conditions than summer climatic conditions [3]. Some studies added bovine serum albumin (BSA) or a mixture of BSA, mucin, and tryptone into virus inoculum used for surface contamination in order to mimic the proteinaceous and organic content of body fluids; this resulted in an extended infectivity period on surfaces of up to 28 days after contamination at 20 °C [16,17]. However, the limitations of this approach are that the content of organic matter is dependent on the type of biological fluids and that fluids contain substances that could be beneficial or harmful for virus survival. These drawbacks led to new approaches to test SARS-CoV-2 stability in biological fluids, such as human nasal mucus, sputum, fecal suspension, and urine [18,19].

In the present study, we determined the potential risk of biological fluids to spread SARS-CoV-2 by testing its stability in nine human biological fluids from which the virus has been previously isolated (nasal mucus, sputum, saliva, tears, feces, and urine) or detected (breast milk, blood, and semen). Generally, SARS-CoV-2 is excreted in biological fluids from infected patients, and can contaminate surfaces and eventually dry on surfaces as the result of water evaporation. The duration of evaporation is dependent on various factors, such as the volume of water and surrounding environmental conditions. Therefore, we evaluated virus stability in various biological fluids using (1) a liquid setting in which the mixture was placed in a sealed tube to prevent evaporation, and (2) a surface setting in which the mixture was completely dried on a stainless steel surface. Both, beneficial and harmful substances concentrate when dried on a surface, and there is little or no activity of microorganisms, broadly active viral inhibitors, or enzymes due to the lack of water in this dried surface setting. In addition, the volume of biological fluids to be excreted and/or discharged varies between biological fluids evaluated in this study. Here, we placed 50 μL of virus-spiked biological fluids in sealed tubes and on stainless steel surfaces to represent a general sized drop of biological fluid. The virus-spiked biological fluids were incubated under indoor and three seasonal conditions that represent U.S. Midwest climate conditions.

Nasal mucus, sputum, and saliva are major components of droplets, which play a primary role in the transmission of SARS-CoV-2. Infectious droplets that are generated by infected individuals can reach the mouth and/or nose of a susceptible individual within a close distance. However, the droplets can also contaminate surfaces and remain infectious for a certain period of time. The potential risk of fomites in SARS-CoV-2 transmission has motivated the investigation of virus stability in biological fluids. A previous study showed that SARS-CoV-2 survived in nasal mucus and sputum for 24 h under both indoor and winter conditions [18].Our data demonstrate that the virus remains infectious for at least 2 days (indoor and summer), 7 days (spring/fall), and 21 days (winter) in nasal mucus and 3 days (indoor), 2 days (summer), 7 days (spring), and 21 days (winter) in sputum; based on the given amount of virus under our experimental design, this indicates a slow virus decay under these conditions. In addition, SARS-CoV-2 survived in saliva for a similar time period under the climatic conditions tested. The disparity in the viral stability might be explained by the different sources of biological fluids, different inoculum titers, and different experimental settings. In the present study, we demonstrated the effect of temperature and relative humidity on virus survival and found that virus stability was significantly extended under spring/fall and winter, but not summer conditions. These results imply the seasonal pattern of SARS-CoV-2 stability in nasal mucus, sputum, saliva, tears, urine, blood and semen. After the first wave of the COVID-19 pandemic took place in spring of 2020, we faced a small rise in the number of new coronavirus infections in June 2020 worldwide, which might be associated with a relaxation of lockdowns and an alleviation of the public’s awareness. However, in the fall and winter of 2020/2021, there has been a drastic resurgence in new daily COVID-19 cases in the northern hemisphere. Along with socioeconomic factors, such as COVID fatigue and more indoor gatherings during colder temperatures, and the emergence of new variants, the seasonality of SARS-CoV-2 stability in biological fluids might be a factor associated with enhanced transmission in fall/winter of 2020/2021.

Despite the tissue and cellular tropism of SARS-CoV-2 in conjunctiva ex vivo [20], the role of tears in transmission has been less obvious due to fewer clinical manifestations and less virus detection/isolation in conjunctival excretion and tears [21,22]. It has been shown that the eye has a mucosal defense system where lysozyme, lactoferrin, lipocalin, and secretory immunoglobulin A are active components in tears where they exert their antimicrobial activity [23]. Lactoferrin has been regarded as a potential inhibitor to SARS-CoV-2 infection because it inhibits SARS-CoV-1 entry into cells by binding to cellular heparin sulfate [24], and SARS-CoV-2 also utilizes heparin sulfate to facilitate the attachment of the spike protein to the angiotensin-converting enzyme 2 receptor [25]. Interestingly, our results show that tears can serve as a viable matrix for the virus, especially in the liquid setting. It is hypothesized that once the virus replicates in conjunctiva tissue and is excreted in tears, it could survive in this environment for an extended time period.

SARS-CoV-2 shedding in feces and urine has been of concern because of the potential transmission via the oro-fecal/naso-fecal route as well as through wastewater. To date, a few studies found infectious SARS-CoV-2 in urine and stool specimens of COVID-19 patients [6,7,8,9]. Our results show that low amounts of virus were detected in human fecal suspensions at 1 hpc, and there was no infectious virus recovered from solid human feces throughout the study, suggesting that the virus is not stable in human feces. It is plausible that infectious virus might survive by suspending stool specimens on a fecal swab or in virus transport medium, and inoculating it onto cells shortly thereafter. In contrast, the virus was stable in urine and survived longer at lower than higher temperatures. These results are comparable to a previous study in which the virus remained infectious for several hours in a fecal suspension and for three to four days in urine at room temperature [19]. In addition, no viable virus was isolated from the steel surfaces contaminated with feces, fecal suspension, and urine at different time points post-contamination in this study. This could be explained by concentrated virucidal substances which could inactivate viable virus during the process of drying. Further analysis is needed to evaluate the potential risk of transmission through wastewater or by-products from sewage, such as biosolids, since most human feces and urine enter the wastewater system to be processed in a sewage treatment plant [26].

SARS-CoV-2 RNAemia has been frequently found in patients suffering a severe course of infection; i.e., the presence of RNA in blood could be a potential factor in predicting clinical outcome [27]. In patients with RNAemia, severe illness is considered the consequence of a dysregulation of the host immune response which is triggered by increased viral load in the serum [28]. SARS-CoV-2 in the blood has been of great interest in terms of virus transmission, as the blood could serve as the source of infection through blood transfusions. However, so far, no evidence supports the transmission of SARS-CoV-2 via blood transfusion [29]. Importantly, our study raises the concern that blood could be involved in the indirect transmission of SARS-CoV-2. Both liquid and dried blood provided highly beneficial conditions to stabilize SARS-CoV-2 infectivity. The high concentration of proteinaceous substances, such as bovine albumin serum, may have a positive effect on virus stability, as observed in a previous study [16].

Similarly, semen has a high concentration of protein contents present in its seminal fluids [30]. This physicochemical property of semen might explain the exceptional virus stability in semen as observed on surfaces, especially under spring/fall and winter conditions. In contrast, a low virus half-life was found for semen in liquid under summer conditions. Previously, it was shown that human immunodeficiency virus is inactivated in semen by at least two different mechanisms: the antiviral activity of H_2_O_2_, which is generated from the oxidation of seminal plasma by diamine oxidase [31], and the intrinsic virucidal activity of cationic polypeptides [32]. These enzymatic and inhibitory activities in semen might account for the faster inactivation of SARS-CoV-2 in the liquid setting, especially under summer conditions.

Vertical transmission of SARS-CoV-2 via breast milk is still being debated [33,34]. Our study showed that infectious virus could be isolated from breast milk only up to 1 dpc in both liquid and surface settings regardless of environmental conditions, implying the low risk of breast milk in fomite transmission. It has been of great interest whether breast milk can act as a vehicle to spread viral diseases. For this purpose, stability studies in breast milk were performed for many viruses, including flavi-, Zika, and hepatitis C viruses. Breast milk has been shown to reduce Zika virus infectivity in a time-dependent manner with its antiviral activity increasing with incubation time [35]. The antiviral activity of breast milk was found to be closely related to the release of free fatty acids by an endogenous lipase, which resulted in the destruction of the envelope layer of hepatitis C virus, and finally the disruption of viral integrity and infectivity [36]. Thus, a similar mechanism resulting in disruption of the viral envelope might explain the instability of SARS-CoV-2 in breast milk.

There are several main limitations of this study. First, no fresh biological fluids were tested in this study. Most biological fluids were collected and frozen at −20 °C. Only blood was kept at 4 °C before use. The pre-storage of biological fluid under frozen conditions before usage in the assay might have an effect on antiviral factors, which was reported for breast milk [35]. Secondly, the duration of the experiment was relatively short (maximum 21 days); therefore, we could not observe the complete disappearance of infectious virus under some of the experimental conditions tested. Nonetheless, we were able to calculate the biological half-life of SARS-CoV-2 in biological fluids and further determined the effects of seasonality. Moreover, biological fluids used in this study were collected from healthy individuals. The physicochemical property of biological fluids could be different in infected and sick individuals, which might result in different virus stability patterns. In addition, the virus stability in biological fluids has been shown to vary greatly between donors [35]; therefore, further in-depth analyses are needed to establish more precise calculations of virus half-life in biological fluids from different donors (healthy and sick) under various conditions. Lastly, we used USA-WA1 isolate, which was isolated from the first U.S. patient in January 2020. However, currently circulating strains have accumulated mutations throughout the genome over time, which might have an impact on the virus stability.

In summary, this study presents the SARS-CoV-2 stability in human biological fluids under indoor and three seasonal conditions. The virus was stable in nasal mucus, sputum, saliva, tears, urine, blood, and semen with t_1/2_ values of 5.23–16.74 h, 2.3–12.57 h, 15.98–54.34 h, and 33.37–235.18 h under indoor, summer, spring/fall, and winter conditions, respectively. The virus survival was significantly longer under either spring/fall or winter than summer conditions, suggesting seasonal effects on the stability of SARS-CoV-2 in biological fluids. Interestingly, we found that SARS-CoV-2 is rather unstable in human feces/fecal suspensions and human breast milk with infectious virus detected only up to 24 h post-contamination. Although fomite transmission still remains poorly defined in the field and is most likely multifactorial, our findings provide new insights into the potential role of biological fluids in SARS-CoV-2 transmission. Furthermore, this knowledge contributes to the development of public health strategies to mitigate the risk of fomites in SARS-CoV-2 transmission, such as cleaning and disinfecting frequently touched surfaces in both indoor and outdoor settings.

## 4. Materials and Methods

Substrates used in this study were nine human biological fluids: nasal mucus, sputum, saliva, tears, feces, urine, breast milk, blood, and semen (Lee Biosolutions, Inc., Maryland Heights, MO, USA). Biological fluids were stored at −20 °C with the exception of blood, which was kept at 4 °C before use. In addition, 10% (*w*/*v*) of fecal suspension was prepared in phosphate-buffered saline. The absence of infectious virus in biological fluids was confirmed by inoculating 50 μL of each biological fluid onto Vero E6 cells and incubating for 4 days. In this study, SARS-CoV-2/USA-WA1/2020 was used, which was isolated from the first U.S. patient in January 2020 and classified as SARS-CoV-2 lineage A and GISAID clade S. The virus stock was prepared and titrated using Vero E6 cells. The virus stock was diluted in DMEM supplemented with 5% FBS to a concentration of 10^7^ TCID_50_/mL, and 5 μL of 10^7^ TCID_50_/mL (i.e., 5 × 10^4^ TCID_50_/5 μL) of virus dilution was added to 45 μL of each respective substrate; a 1:10 dilution was used as to not over-dilute the biological fluids. The mixture was placed in a sealed tube to test virus stability in a liquid and onto stainless steel (1/2 inch in diameter and 16 gauge thickness, Metal Remnant Inc., Salt Lake City, UT, USA) in a 12-well plate to test virus stability on a surface. For nasal mucus, sputum, and feces, 0.10 g to 0.15 g of each substrate was placed in a tube or on stainless steel, spiked with the same amount of virus, and gently mixed with a pipette tip. As a positive control for each setting, we used the same volume of Dulbecco’s Modified Eagle’s Medium (DMEM) supplemented with 5% fetal bovine serum (FBS) instead of the substrate. For a negative control, 50 μL of DMEM supplemented with 5% FBS was placed in the tube or on steel. The virus-spiked substrates on stainless steel were air-dried for 4 h inside a biosafety cabinet at room temperature (approximately 21–23 °C) to recapitulate dried infectious biological material on a non-porous surface. The liquid virus in the sealed tube and the dried virus on the stainless steel were incubated in an environmental chamber (Humidity and Temperature Stability Test Chambers, Nor-Lake Scientific, Hudson, WI, USA) under four different environmental conditions: 21 °C/60% RH, 25 °C/70% RH, 13 °C/66% RH, and 5 °C/75% RH, which simulates indoor, summer, spring/fall, and winter conditions, respectively [3]. The door of the chamber was opened only when placing and removing samples in order to minimize the change of controlled temperature and humidity conditions; opening of the door resulted in a transient fluctuation of RH, which recovered within a few minutes. The infectious virus was recovered at the respective time points in 2 mL DMEM supplemented with 5% FBS and filtered through a 0.45 μm syringe filter (TPP, Trasadingen, Switzerland) (Table 2). To determine virus titer, 10-fold serial dilutions were prepared and inoculated onto Vero E6 cells. The presence of cytopathic effects, such as cell rounding and detachment, were recorded at 4 days post-inoculation to calculate TCID_50_/mL using the Reed–Muench method. The assays were performed in triplicate. Log-transformed virus titers in nasal mucus, sputum, saliva, tears, urine, blood, semen, and positive control were incorporated to estimate a simple linear regression in Prism 9 (GraphPad, San Diego, CA, USA). The first time points for analysis were 1 hpc for liquid setting and 4 hpc for surface settings, and virus titers at time points when at least one replicate was positive were incorporated for the analysis. Half-life (t_1/2_) was calculated as a −log_10_(2)/slope. To determine the seasonal pattern of stability, one-way analysis of variance (ANOVA) was used to compare the slopes of linear regression under summer, spring/fall, and winter conditions according to the software’s instruction. Multiple pairwise comparisons using Tukey’s adjustments were subsequently performed to identify significant differences between two conditions and adjusted p values are shown. Significant differences between liquid and surface settings were tested using default analysis, which is compatible to analysis of covariance in GraphPad Prism 9. Raw data and the virus titer calculations are available upon request.

## Figures and Tables

**Table 1:** Half-life of SARS-CoV-2 in human biological fluids under indoor, summer, spring/fall, and winter conditions.

Environmental Condition	21 °C/60% RH Indoor		25 °C/70% RH Summer		13 °C/66% RH Spring/Fall		5 °C/75% RH Winter	
Setting	Liquid	Surface	Liquid	Surface	Liquid	Surface	Liquid	Surface
Nasal mucus	5.23 (4.03, 7.47) ^1^	6.77 (4.57, 13.01)	4.59 ^2^ (3.66, 6.17)	2.58 ^2^ (2.02, 3.59)	21.74 (15.61, 35.76)	18.15 (14.66, 23.83)	53.94 (44.82, 67.71)	38.55 (30.42, 52.62)
Sputum	8.69 ^2^ (7.11, 11.17)	14.9 ^2^ (10.72, 24.37)	3.68 (2.59, 6.37)	5.55 (4.42, 7.45)	21.94 ^2^ (16.05, 34.66)	37.03 ^2^ (27.74, 55.67)	33.37 ^2^ (22.75, 62.56)	76.4 ^2^ (60.48, 103.7)
Saliva	7.89 ^2^ (6.38, 10.37)	12.69 ^2^ (10.36, 16.38)	6.98 (5.19, 10.65)	6.44 (4.87, 9.5)	15.98 ^2^ (12.51, 22.12)	34.17 ^2^ (27.05, 46.28)	55.16 (44.28, 73.15)	69.25 (61.3, 79.57)
Tears	15.1 ^2^ (12.29, 19.56)	8.3 ^2^ (7.09, 10)	11.06 ^2^ (8.91, 14.58)	3.53 ^2^ (2.93, 4.45)	29.34 (25.75, 34.07)	24.22 (20.14, 30.38)	121.83 (91.19, 183.44)	106.82 (84.44, 145.43)
Urine	11.41 (9.69, 13.88)	N/A ^3^	7.89 (6.74, 9.5)	N/A ^3^	54.34 (36.76, 103.8)	N/A ^3^	57.73 (49.06, 70.15)	N/A ^3^
Blood	16.74 (11.64, 29.83)	16.57 (13.1, 22.53)	12.57 (9.58, 18.3)	10.75 (9.16, 13)	39.25 (28.03, 65.26)	48.4 (35.79, 74.6)	102.04 ^2^ (71.93, 175.63)	235.18 ^2^ (144.52, 631.75)
Semen	7.48 (5.76, 10.65)	9.51 (7.46, 13.1)	2.3 ^2^ (1.75, 3.33)	5.9 ^2^ (4.75, 7.78)	17.69 ^2^ (13.96, 24.14)	41.24 ^2^ (29.31, 69.31)	57.81 ^2^ (47.77, 73.23)	91.64 ^2^ (75.24, 117.22)
Positive control	15.85 ^2^ (11.81, 24.12)	7.88 ^2^ (6.48, 10.05)	7.48 ^2^ (6.7, 8.46)	2.57 ^2^ (2.21, 3.08)	48.95 ^2^ (35.25, 80)	21.5 ^2^ (17.25, 28.56)	176.66 ^2^ (109.78, 451.39)	79.64 ^2^ (63.6, 106.48)

^1^ Half-life (95% confidence interval) in hours. ^2^ Significant difference (*p* < 0.05) between liquid and surface settings under a respective condition. ^3^ N/A: Not available. RH: Relative Humidity.

**Table 2:** A series of time points at which the virus was collected and titrated on Vero E6 cells after incubation under indoor or different seasonal conditions.

Environmental Condition	21 °C/60% RH/Indoor, 25 °C/70% RH/Summer	13 °C/66% RH/Spring/Fall	5 °C/75% RH/Winter
Liquid setting	1 h post-contamination (hpc), 7 hpc, 1 day post-contamination (dpc), 2 dpc, and 3 dpc	1 hpc, 1 dpc, 3 dpc, 5 dpc, and 7 dpc	1 hpc, 1 dpc, 3 dpc, 6 dpc, 10 dpc, 15 dpc, and 21 dpc
Surface setting	4 hpc, 8 hpc, 1 dpc, 2 dpc, and 3 dpc	4 hpc, 1 dpc, 3 dpc, 5 dpc, and 7 dpc	4 hpc, 1 dpc, 3 dpc, 6 dpc, 10 dpc, 15 dpc, and 21 dpc

## Data Availability

Raw data and the virus titer calculations are available upon request.
